# Supplementary figures and images for: Imaging of neural oscillations with embedded inferential and group prevalence statistics
Source: PLoS Comput Biol. 2018 Feb 6;14(2):e1005990. doi: 10.1371/journal.pcbi.1005990 (PMC5815621; doi:10.1371/journal.pcbi.1005990)

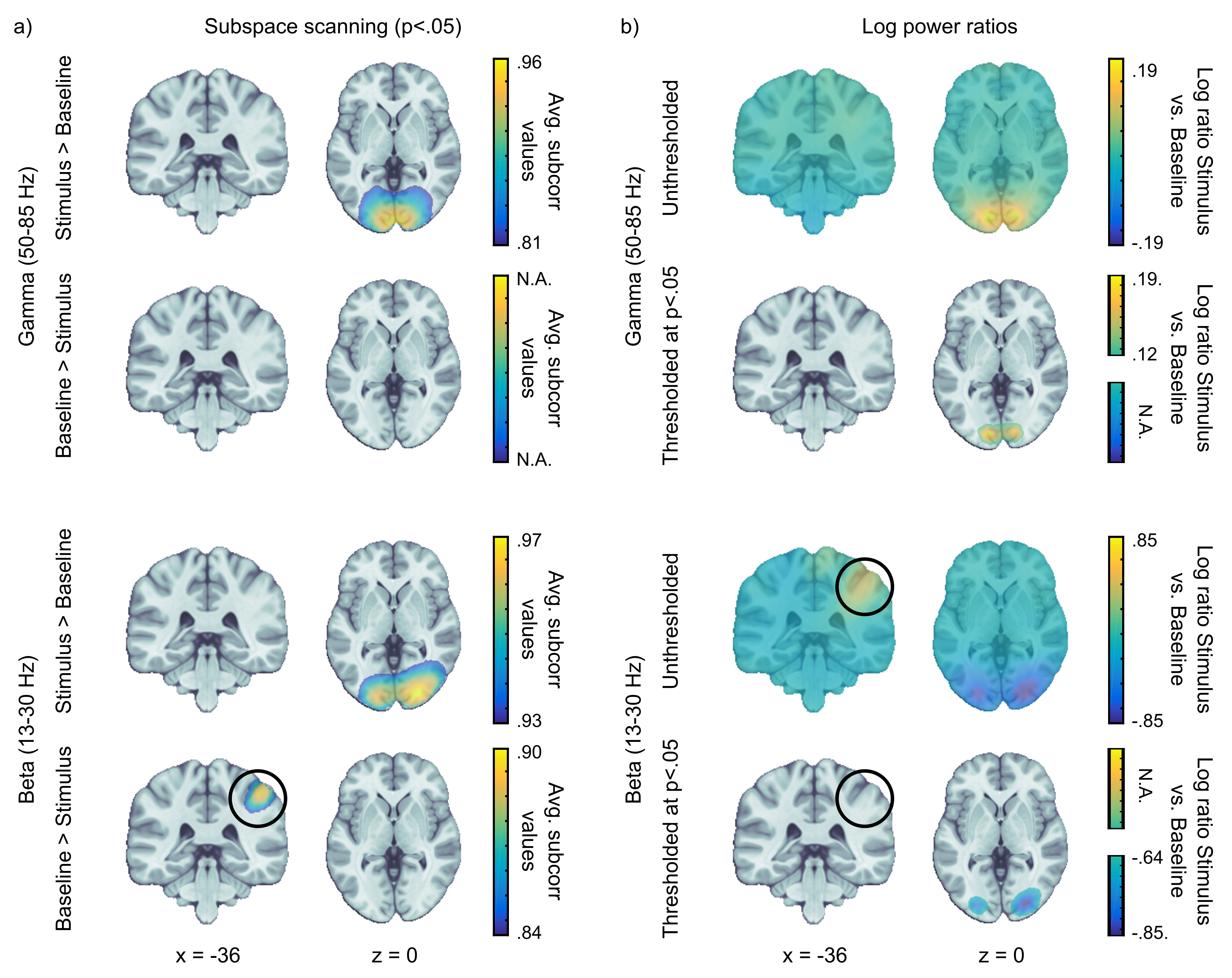

Supplement: S1 Fig — All analysis parameters are equivalent as described in the main part of the paper. (TIF) [file pcbi.1005990.s001.tif]

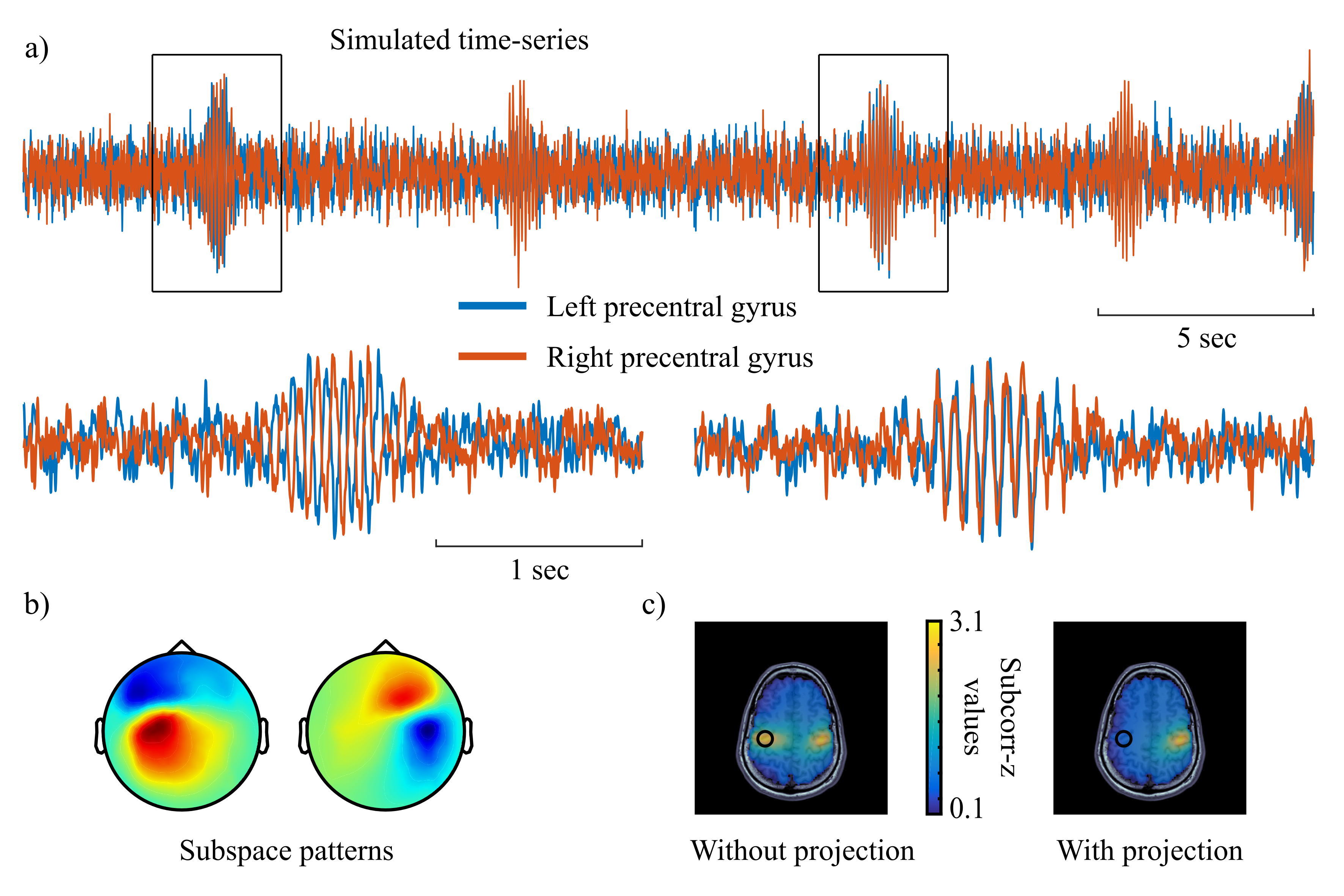

Supplement: S2 Fig — We simulated 300 seconds of source signals in 68 regions of interest with a 1/f spectral profile (same as Fig 7 in the main text). We added oscillatory bursts to four of these ROIs (precentral left/right and rostralmiddlefrontal left/right) modelled by a Morlet wavelet at 12 Hz with a FWHM of 250 ms. These were set to occur at the same time in left/right homologous regions in 50% of the time, such that the resulting amplitude correlation in the alpha band (8-13 Hz) was above 0.4. Panel a) shows such a signal pair. The phase delay between these oscillatory bursts was uniformly random (see enlarged signal parts). We then performed an iES analysis as shown in Fig 8 of the main text (functional connectivity) with a left precentral seed. We can see in panel b) the obtained subspace patterns for a threshold of r > .4. Note that we obtained two spatial patterns that corresponded to the leadfields of the two regions correlated to the seed (precentral left/right). Panel c) shows that the iES maps revealed the correlated contralateral source with or without the projection step explained in the main text. We can also see that only the correlated sources were present in the map (precentral left/right) and not the sources that had oscillatory bursts in this frequency band (rostralmiddlefrontal left/right) but were not correlated to the seed. (TIF) [file pcbi.1005990.s002.tif]

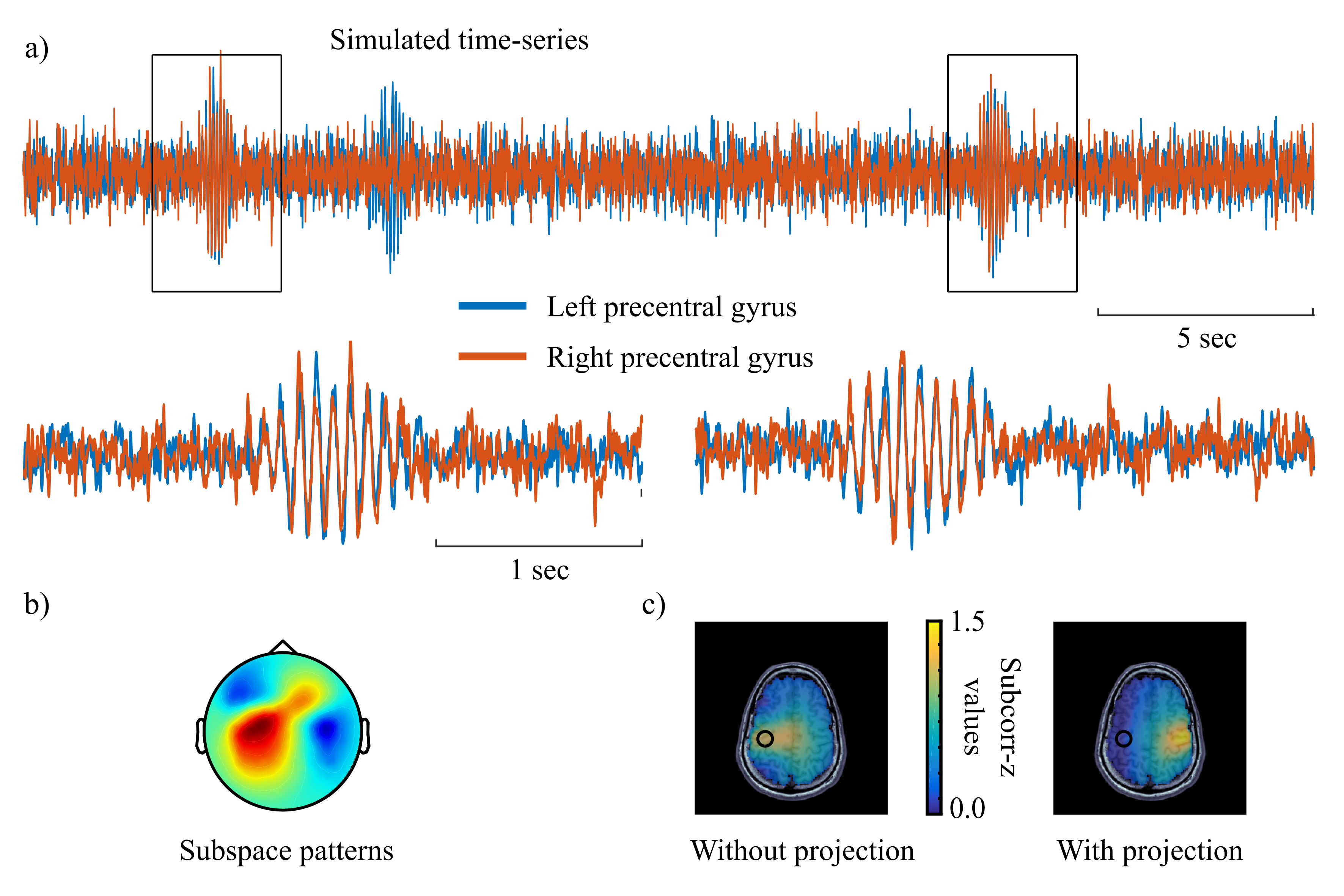

Supplement: S3 Fig — We simulated the same setup as in S2 Fig with the difference that the co-occuring oscillatory bursts in left/right homologous regions had zero phase delay (see enlarged signal parts in panel a). Even though the amplitude correlations were still above 0.4, a linear combination of the leadfields of both sources was captured in one subspace pattern (panel b). This is because the decomposition produces subspace patterns whose corresponding signals are orthogonal. We see in panel c) that the iES map without projection applied did not reveal the contralateral correlated region. However when projecting out the seed topography, the peak in the contralateral region was revealed, as expected. This illustrates the difference between iES and other approaches that orthogonalize signals, which would remove the correlated oscillatory bursts in this scenario. (TIF) [file pcbi.1005990.s003.tif]

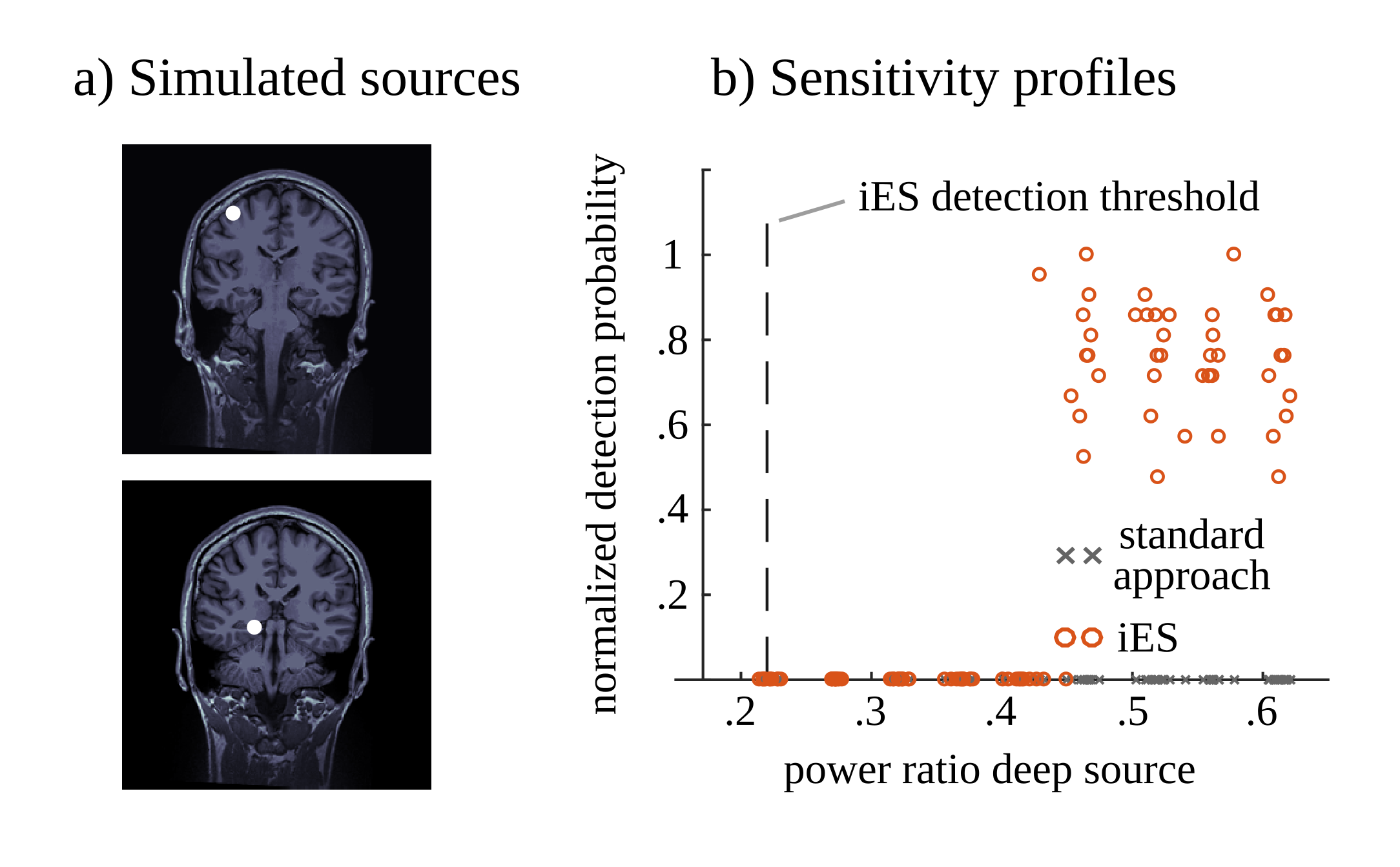

Supplement: S4 Fig — a) Two sources of interest (precentral left and parahippocampal left, according to the Desikan-Killiany atlas, [22]) targeting pre-specified power ratios are embedded in background brain noise composed of 1/f signals evenly distributed across 66 locations. The superficial source is simulated at a fixed power ratio (narrowband vs. broadband power) of 0.6, the power ratio of the deep source is varied between 0.2 and 0.6. b) Normalized detection probability is calculated as in Fig 7 and shows that, while the scenario is more challenging for iES (higher power ratio of the deep source is needed to detect both sources), it outperforms MNE, which did not detect both sources in this scenario. (TIF) [file pcbi.1005990.s004.tif]
